# Similar effects of high-fructose and high-glucose feeding in a *Drosophila* model of obesity and diabetes

**DOI:** 10.1371/journal.pone.0217096

**Published:** 2019-05-15

**Authors:** Laura Palanker Musselman, Jill L. Fink, Thomas J. Baranski

**Affiliations:** Division of Endocrinology, Metabolism and Lipid Research, Department of Medicine, Washington University School of Medicine, St. Louis, Missouri, United States of America; CINVESTAV-IPN, MEXICO

## Abstract

As in mammals, high-sucrose diets lead to obesity and insulin resistance in the model organism *Drosophila melanogaster* (called *Drosophila* hereafter). To explore the relative contributions of glucose and fructose, sucrose’s component monosaccharides, we compared their effects on larval physiology. Both sugars exhibited similar effects to sucrose, leading to obesity and hyperglycemia. There were no striking differences resulting from larvae fed high glucose versus high fructose. Some small but statistically significant differences in weight and gene expression were observed that suggest *Drosophila* is a promising model system for understanding monosaccharide-specific effects on metabolic homeostasis.

## Introduction

Much ado has been made about the negative health effects of high-fructose corn syrup. A number of laboratory studies have shown that fructose is worse for health than its structural isomer glucose [1] and references therein]. Controlled clinical studies have shown increased hyperglycemia and reduced insulin sensitivity during fructose feeding, compared with glucose feeding, although the mechanisms downstream are not well-understood [2]. Both fructose and glucose are six-carbon hexose sugars and can be catabolized via glycolysis or metabolized to store as fat. One difference between them is the liver can take on fructose preferentially and suffers metabolic overload as a result [3]. Glucose and fructose are both common in the diet. High-fructose corn syrup, which is typically 55% fructose, also contains 42% glucose [4]. Cane or beet sugar is sucrose, a disaccharide of fructose and glucose that is just as sweet as high-fructose corn syrup. Sucrose is digested by intestinal sucrase to deliver a 50:50 ratio of fructose to glucose to peripheral tissues, nearly the same ratio as in high-fructose corn syrup. Previous studies showed that *Drosophila melanogaster* (called *Drosophila* from now on) fed high-sucrose diets (24–34% sucrose) developed type 2 diabetes-like pathophysiology including increased triacylglycerides, hyperglycemia, and insulin resistance, compared with control diets [5% sucrose, [5–11]]. We set out to understand how different sugars might be metabolized differently *in vivo* using this *Drosophila* model.

In many cells, glucose is readily converted into fructose and vice versa via phosphohexose isomerase or via three-carbon catabolic intermediates [12]. When cellular energy is low, the cell will oxidize either monosaccharide via glycolysis to make ATP. When energy is high, the cell shunts these monosaccharides into polysaccharides like glycogen or amylose, into the lipogenic pathway, or into glycoprotein synthesis [12,13]. In the circulation, however, glucose and fructose meet different fates, at least in mammals. Glucose is taken up by many peripheral tissues including liver, adipose, muscle, and heart, where its metabolism can be attenuated by reducing glucose uptake or by feedback inhibition of phosphofructokinase under high-energy conditions [12]. An important feature of glucose in the liver is that it can be sequestered or produced there and released to increase the blood’s glucose supply [14]. In contrast with glucose, fructose is more likely to be catabolized by the liver, bypassing phosphofructokinase and potentially overloading glycolysis and downstream pathways [1]. Dietary fructose, therefore, favors hepatic lipogenesis more than glucose [3,15].

The *Drosophila* fat body serves as both the fly adipose and liver. This tissue specializes in fat storage and metabolism, increasing in lipid content when the fly is overfed and decreasing in fat storage when the fly is starved [16]. In previous studies, we found that fat bodies from larvae subjected to high-sucrose feeding developed insulin resistance and accumulated potential lipotoxins including free fatty acids and triacylglycerides [17–19]. Fat bodies also control systemic metabolic homeostasis by endocrine mechanisms including cytokines and adipokines [20–23]. Therefore, fat body physiology reflects metabolic regulatory mechanisms of the liver and adipose in mammals.

Because of the notable effects of fructose and glucose in humans, we exploited a high-sugar-induced type 2 diabetes model to look for conserved differences between fructose and glucose during overnutrition in *Drosophila*. By directly comparing glucose and fructose-reared larvae, we detected only a few minor differences that suggest fructose may be worse for flies than glucose. Overall, high fructose and high glucose diets produced similar negative effects on fly physiology.

## Results

In previous studies, we tested dose responses to various sugars and saw similar effects on developmental delay [5]. To extend those studies, we chose glucose and fructose, two monosaccharides frequently ingested by humans. To enable comparison with our 0.15 M sucrose control diet (approximately 5% sugar, as in many fly diets), we used 0.3 M fructose or 0.3 M glucose diets as our controls (CFD or CGD, respectively). Because previous studies had used 1 M sucrose, a disaccharide, we prepared food containing 2 M of each monosaccharide. 2 M fructose or glucose, however, proved to be highly toxic to larvae, especially in one of our wild-type control genotypes. Consequently, we reduced sugar concentrations to settle on a high-sugar dietary concentration of 1.7 M fructose (high fructose diet, HFD) or 1.7 M glucose (high glucose diet, HGD). The control genotype larvae were the offspring of the cross between *w*^*1118*^ males and *UAS-Dcr2; r4-GAL4* females, which we have characterized extensively. These animals are phenotypically normal and we have generated a large amount of data using them [17–19,24].

**Fig 1:**
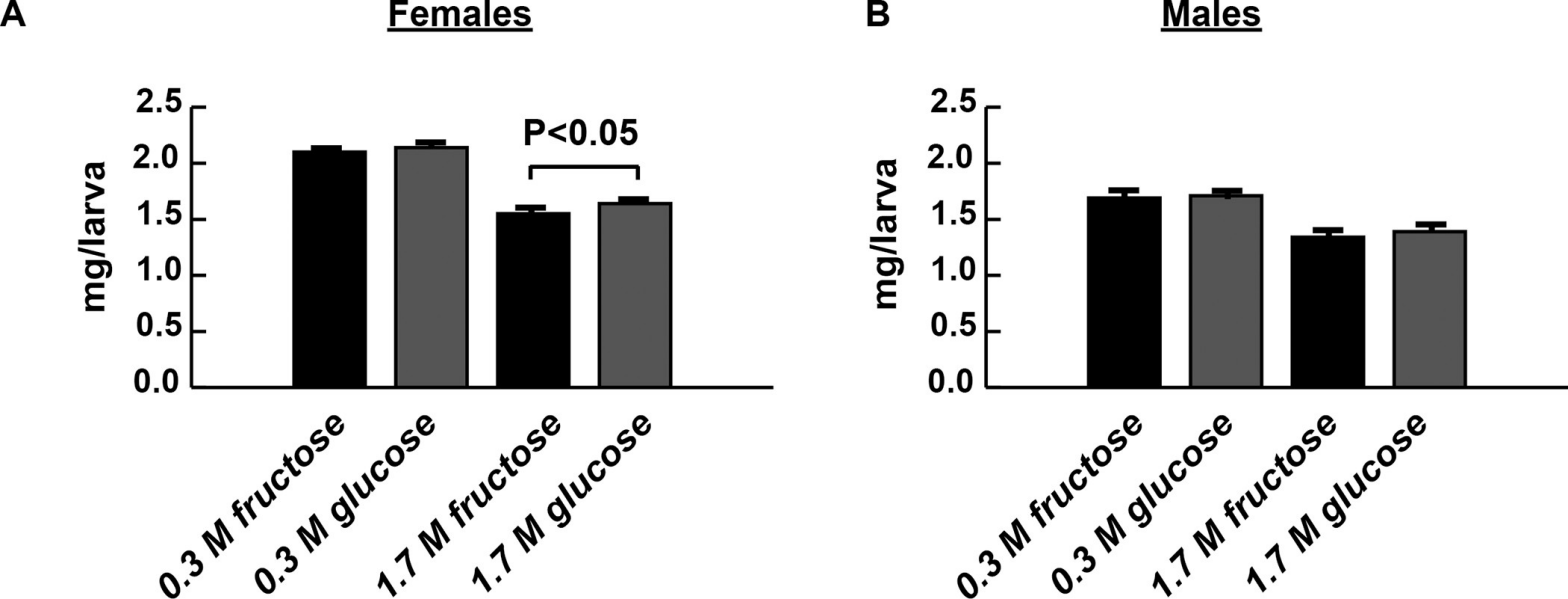
High-sugar diets reduce weight, compared with control diets. 1.7 M monosaccharide diets reduced wandering third instar larval weights compared with either 0.3 M monosaccharide diet, all with adjusted P < 0.001 in females (A) or males (B) using a one-way ANOVA followed by Tukey’s multiple comparisons test. HFD modestly but significantly reduces weight by 5.5% compared with HGD rearing in female larvae (A). A 3.6% reduction in male weight between HGD and HFD was not statistically significant (B). n = 17–30 biological replicates. Error bars represent the S.E.M.

Insulin resistance in *Drosophila* is accompanied by decreased weight and increased hemolymph glucose and body fat content in both larvae and adults [25–27]. High sucrose diets also elicit all of these phenotypes [5,6,28], so we tested HFD and HGD larvae for insulin resistant phenotypes. Because of the developmental delay, we isolated third instar larvae at the same developmental stage, when fully developed larvae wander from the food in preparation for metamorphosis, to enable direct comparison. Rearing on HFD and HGD reduced wandering third instar larval weights compared with CFD and CGD rearing (Fig 1A and 1B). While the two control diets did not produce significant differences in weight, HFD-rearing did produce female larvae of significantly smaller weight, compared with the HGD (Fig 1A). High fructose reduced weight by 26.1% in females and 20.7% in males, whereas high glucose reduced weight by 23.5% and 18.7% in females and males, respectively.

**Fig 2:**
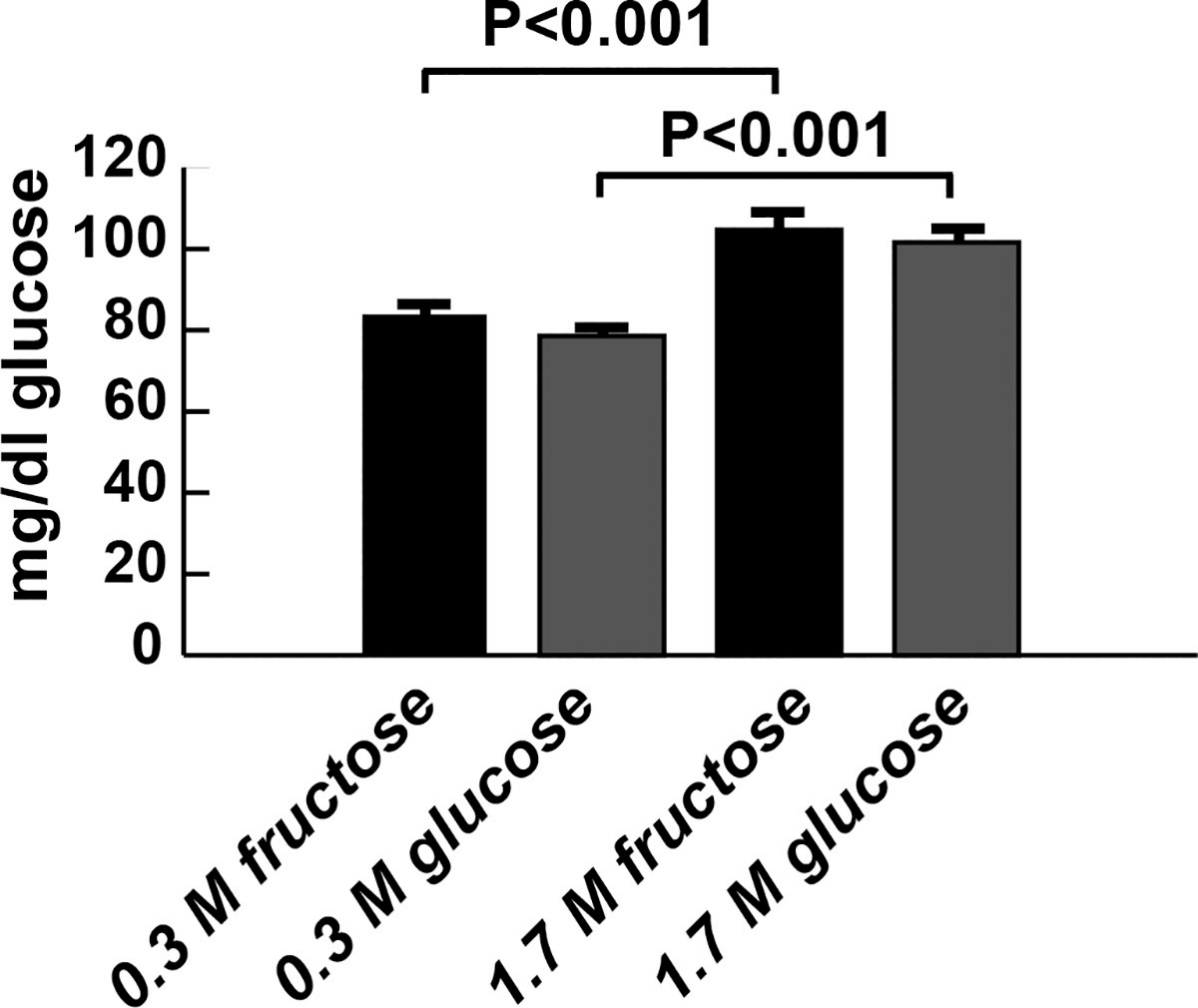
Chronic high monosaccharide diet feeding leads to hyperglycemia. Both HFD and HGD led to hyperglycemia, compared with CFD and CGD. n ≥ 36 biological replicates. Significance was determined using a one-way ANOVA followed by Tukey’s multiple comparisons test. Error bars represent the S.E.M.

Insulin resistance is also accompanied by hyperglycemia, or increased blood glucose concentrations. Therefore, we quantified glucose levels in the *Drosophila* blood, known as hemolymph. Both HFD and HGD increased hemolymph glucose concentrations compared with CFD and CGD (Fig 2). However, no difference was seen between fructose and glucose on either 0.3 M diets (P = 0.2722) or 1.7 M diets (P = 0.6101)

**Fig 3:**
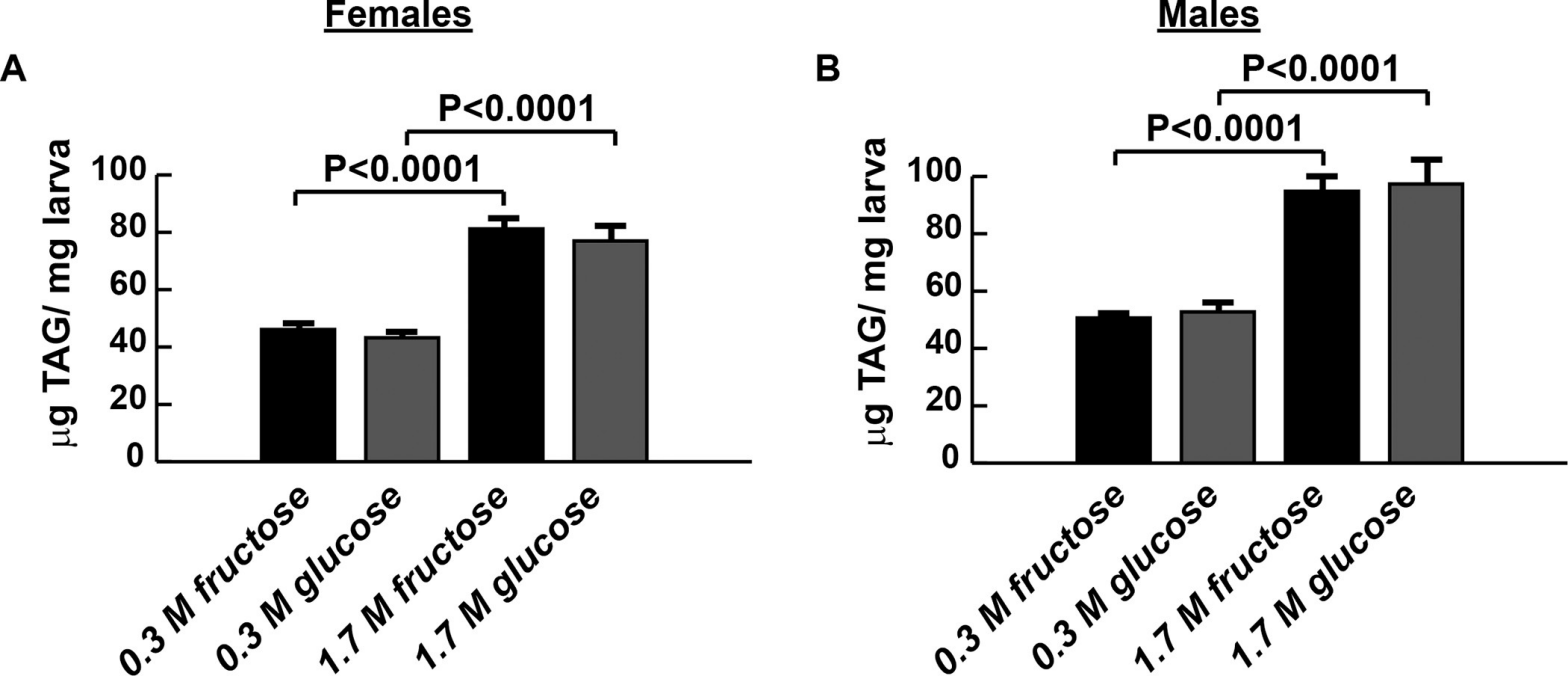
Chronic high monosaccharide diet feeding leads to obesity. Both HGD and HFD 1.7 M sugar diets increased TAG concentration in wandering third instar larvae, compared with respective controls. There were no differences in TAG between fructose and glucose diets. n = 17–30 biological replicates. Significance was determined using a one-way ANOVA followed by Tukey’s multiple comparisons test. Error bars represent the S.E.M.

Overnutrition is associated with increased fat storage or obesity. Therefore, we quantified whole animal triacylglycerides (TAG) in larvae reared on CFD, CGD, HFD, and HGD. Like the high-sucrose diet, both HFD and HGD increased TAG concentrations (Fig 3A and 3B). As with hemolymph glucose, we saw no differences between fructose-fed and glucose-fed larval TAG content on either type of diet.

**Fig 4:**
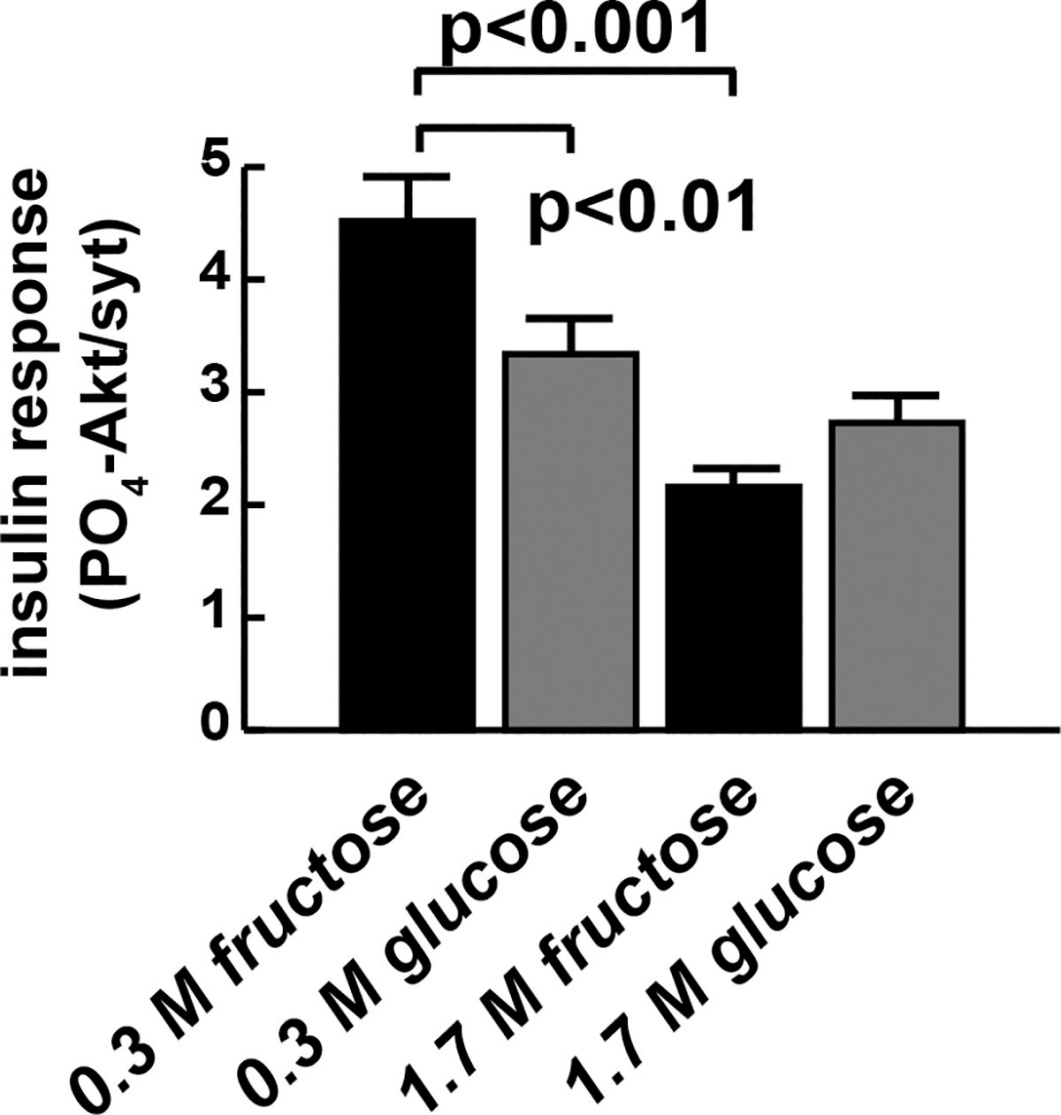
Fructose reduces insulin signaling more than glucose. Chronic HFD (1.7 M fructose) feeding significantly increases insulin resistance, measured by Akt phosphorylation response, compared with CFD feeding (0.3 M fructose). A one-way ANOVA followed by Tukey’s multiple comparisons test was used to compare diets. n = 15 for CGD. n = 14 for HGD. n = 10 for CFD and HFD. Error bars represent the S.E.M.

Insulin resistance often correlates with reduced molecular signaling activity. Insulin receptor activation leads to a cascade of phosphorylation events including the kinase Akt (also called PKB). Phosphorylation of *Drosophila* Akt at serine 505 is a reliable indicator of insulin signaling [29]. Phospho-Akt, in turn, exerts a negative effect on FOXO, a negative regulator of insulin-dependent growth and glucose uptake and catabolism. We quantified PO_4_-Akt in CFD, CGD, HFD, and HGD larval fat bodies stimulated with 1 μM recombinant human insulin as a way to measure insulin sensitivity (Fig 4). The most dramatic effects again resulted from HFD feeding: HFD caused a 50% reduction in fat body insulin sensitivity, compared with CFD-reared larvae (Fig 4). In contrast, HGD feeding did not have the same effect when compared with CGD (p > 0.5). To our surprise, the CGD led to reduced fat body insulin sensitivity, compared with the CFD. By contrast, the HFD was no more sensitive to insulin stimulation than the HGD (Fig 4). These data are not easily explained but are consistent with a model where fructose and glucose have distinct effects on insulin signaling in the *Drosophila* fat body.

**Fig 5:**
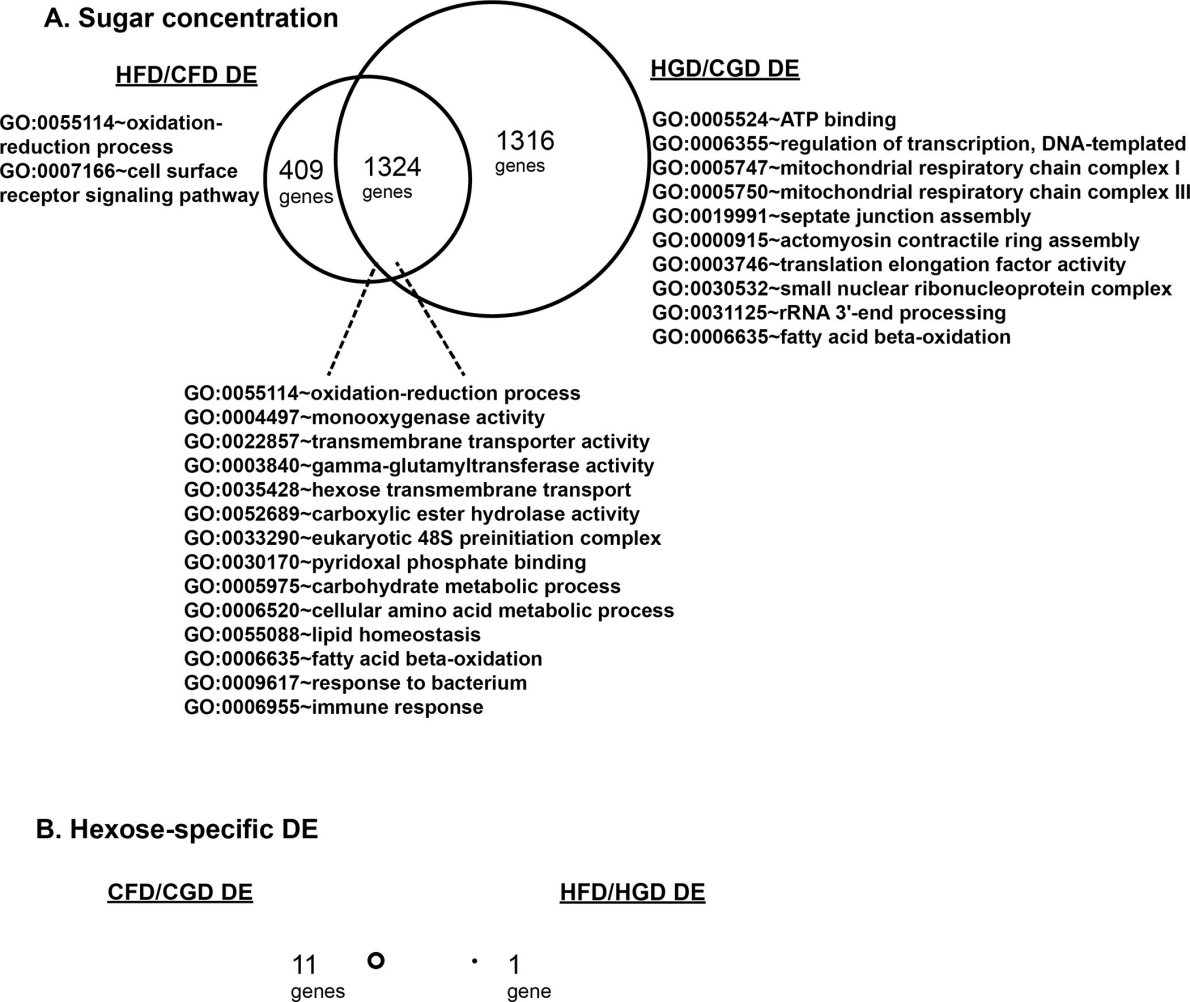
High-sugar feeding leads to effects on gene expression. (A) Both high-sugar diets (HFD, HGD) lead to significant changes in gene expression, compared with control (CFD, CGD) diets. The enriched gene ontology (GO) categories affected included many related metabolic pathways. (B) Hexose-specific gene expression profiles contained a total of 12 differentially-expressed genes (Table 1). No GO categories were enriched in these gene lists. Three biological replicates were sequenced for each sample type and EdgeR was used to select differentially expressed genes using the negative binomial model exact test.

To look more closely at target tissue differences between the two sugar diets, we used RNA-seq to characterize gene expression in the larval fat body after rearing on all four diets. Fat bodies were collected from female wandering third instar larvae, RNA isolated, and Illumina Hi-Seq and differential expression analysis done by the Washington University Genome Technology Access Center. As in previous studies comparing control and high sucrose fat body, many genes were differentially expressed (DE) between control and high monosaccharide diets. For fructose, EdgeR identified 1733 DE genes between CFD and HFD, with 2641 DE genes between CGD and HGD (Fig 5, Supplemental data at GEO accession GSE121059). More than half (1324) of sugar-DE genes overlapped between the fructose and glucose datasets (Fig 5A), all of which showed the same direction of change (up- or down-regulated by high sugar). Using these data, we found that only a small number of genes were significantly DE between glucose and fructose feeding in the fat body (Fig 5, Table 1). Eleven genes were DE between CFD and CGD fat bodies. Three of the DE genes, *Sgs7* (*CG18087*), *IM3* (*CG16844*), and *CG42798* were also DE in independent studies profiling InR targets in the fat body [24]. There was an interesting relationship between glucose and insulin signaling: each gene had the same response in high sucrose-fed *InR* RNAi (relative to wild-type) as it did to glucose (relative to fructose, Table 1) with constitutively active InR producing the opposite change. Thus, while both CFD and CGD appear to provide a healthy diet for flies, they do exhibit significant differences at the gene expression level in the fat body.

**Table 1:** Differential expression between all-glucose and all-fructose diets. RNA-seq and differential expression analysis identified twelve DE genes with p<0.0001 and a false discovery rate under 5% for 5% w/v CFD versus CGD monosaccharide diets. For HFD and HGD (30.6%w/v monosaccharide), only one gene, *CG6602*, significantly differed in fat body expression level. Four of the twelve differentially expressed genes were also regulated by InR [24].

**Gene ID**	**Gene name**	**Gene description**	**P value**	**FDR**	**CGD/CFD**	**InRi FC- HS**	**InRCA FC**
*CG33128*	*CG33128*	putative aspartic-type endopeptidase	2.24E-05	0.04163	-9.2678		
*CG5767*	*CG5767*	DUF725 family	3.43E-05	0.04163	-4.66179		
*CG18087*	*Sgs7*	Salivary gland secretion 7	7.33E-06	0.022108	-4.40159	-104.13091	5.842893
*CG15404*	*CG15404*		5.66E-06	0.022108	-4.07992		
*CG7606*	*CG7606*		2.98E-05	0.04163	-3.83348		
*CG42798*	*CG42798*		3.8E-05	0.04163	-3.21595	-3.4530228	3.878016
*CG11720*	*Sgs3*	Salivary gland secretion 3	1.34E-05	0.032438	-2.8747		
*CG7178*	*wupA*	wings up A; troponin	3.36E-05	0.04163	-2.72439		
*CG33282*	*CG33282*	SLC2A family; putative glucose transporter	5.8E-06	0.022108	2.417307		
*CG13075*	*CG13075*	chitin metabolism	2.49E-06	0.022108	3.852006		
*CG16844*	*IM3*	Immune induced molecule 3	3.56E-05	0.04163	7.837073	377.902483	-3.26191
**Gene ID**	**Gene name**	**Gene description**	**P Value**	**FDR**	**HGD/HFD**	**InRi FC- LS**	**InRCA FC**
*CG6602*	*CG6602*		2.99E-09	2.88E-05	5.809633	5.96770702	-2.5801

Comparing HFD to HGD, only one gene was differentially-expressed, *CG6602* (Fig 5, Table 1). This gene was expressed at significantly higher levels in HGD-fed fat bodies, compared with HFD fat bodies. Interestingly, *CG6602* expression did not differ between CFD and CGD (Supplemental data at GEO dataset GSE121059). Therefore, there were no overlapping genes between the two monosaccharide-dependent gene sets (Fig 5B). We observed no differential expression in *CG6602* between control and high sucrose fat bodies [17,18] and *CG6602* was differentially expressed in control-fed but not high-sugar-fed *InR* RNAi fat bodies [24]. Like some of the DE genes detected comparing CFD and CGD monosaccharide diets, we noted that this gene had a similar response to InR RNAi as it did to glucose: both increased *CG6602* expression. Conversely, constitutively active InR decreased *CG6602* expression (Table 1). Although not much is known about *CG6602*, its expression was also reduced 50% by loss of Sir2 in whole adult flies [30]. Therefore, we chose to target *CG6602* in the fat body using transgenic RNA interference (RNAi). We predicted that *CG6602* might be upregulated to protect larvae from adverse effects of the HGD.

**Fig 6:**
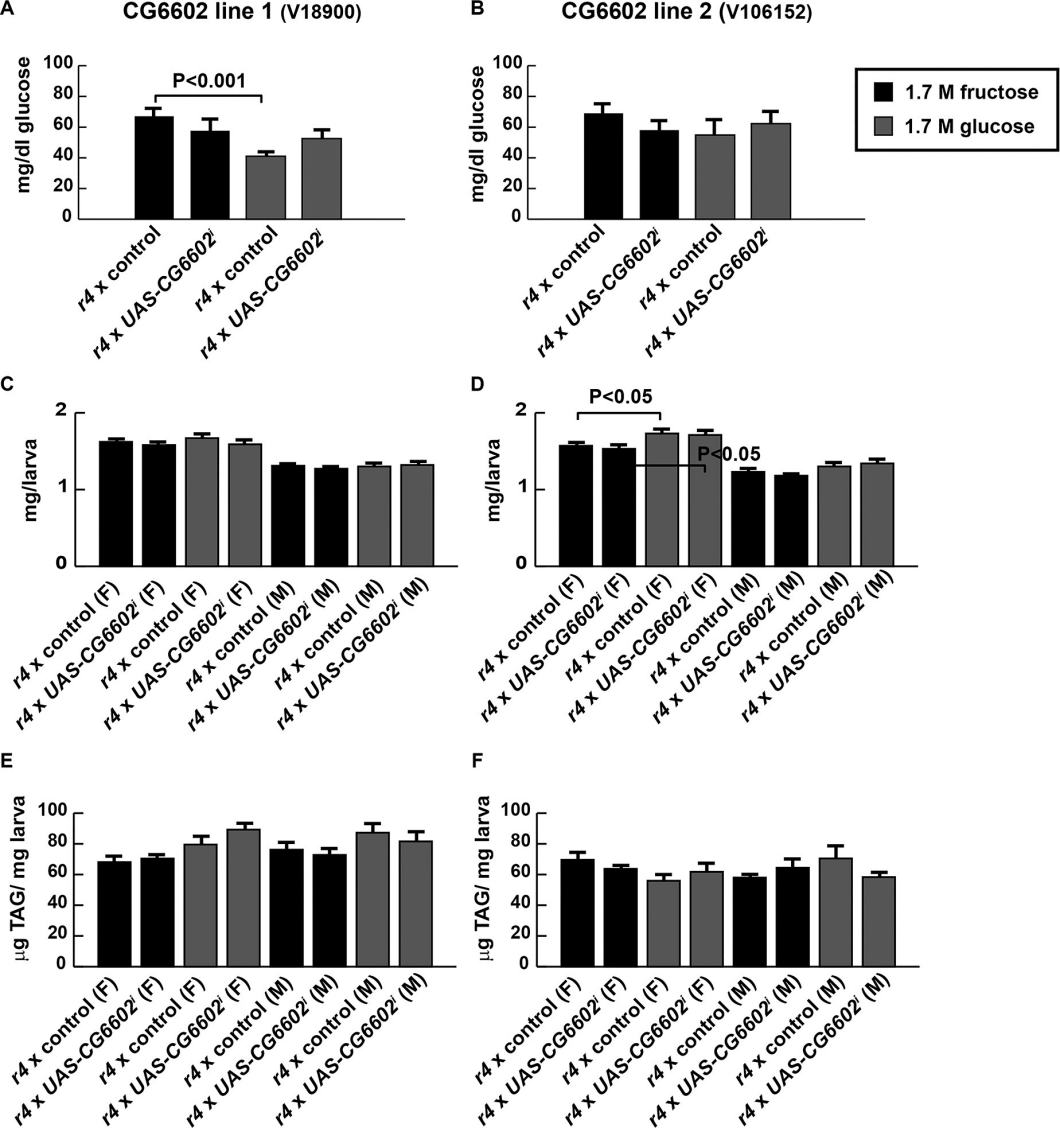
Minimal effects of *CG6602* knockdown on high-sugar-induced phenotypes. (A) CG6602 RNAi did not significantly affect hemolymph glucose concentrations in wandering third instar larvae, although fructose and glucose were different in one control genotype. (B) No significant differences were observed, although similar trends of sugar and CG6602 dependence occurred in both genotypes. (C) (D) No effects on larval weights were observed with *CG6602* knockdown. (E) (F) No effects on larval TAG were observed with *CG6602* knockdown. n ≥ 5. A one-way ANOVA followed by Tukey’s multiple comparisons test was used to compare groups. Error bars represent the S.E.M.

Two *UAS*-RNAi transgenic lines, *CG6602i*^*18900*^ and *CG6602i*^*106152*^ [31], were used to target this gene in the fat body using an r4-GAL4 driver [32] using the GAL4/UAS bipartite transgenic system [33]. A *UAS-Dcr2* transgene was also used to increase the efficacy of RNAi [31]. In parallel, lines of the genetic background into which each transgene was inserted (VDRC stocks 60000 and 60100, described in methods) were crossed to driver lines to serve as controls. We compared hemolymph glucose, TAG, and weight at larval maturity for each of the four genotypes, two *CG6602* RNAi and two controls, on the HFD and HGD. In general, our observations showed little difference in the responses to fructose and glucose. More severe phenotypes in high-fructose as compared with high-glucose diets occasionally reached statistical significance (6A, 6D). None of the phenotypes tested were dramatically affected by *CG6602* RNAi (Fig 6A–6F). Reducing *CG6602* had non-significant effects on hemolymph glucose on either high-sugar diet (Fig 6A and 6B). Neither growth nor TAG content was affected by *CG6602* knockdown in larvae reared on either diet (Fig 6C–6F).

## Discussion

Our results extend previous studies to investigate the effects of monosaccharide feeding in *Drosophila* larvae. In our model, high fructose is not much worse for flies than high glucose with respect to any phenotypes. Both HFD and HGD induced hyperglycemia and obesity along with hundreds of changes in gene expression. There were few changes in gene expression between glucose and fructose-containing diets. High fructose and high glucose diets led to comparable degrees of obesity and diabetes. Overall, HFD and HGD phenotypes were much like those on a high sucrose diet [5]. There were only minor differences, leading us to hypothesize that monosaccharide composition is not critical for larvae; rather, physiology depends on sugar concentration.

Recent studies have also explored the role of different monosaccharides in *Drosophila* [34–37]. The Lushchak group noted a range of effects of fructose and glucose on *Drosophila* growth, lifespan, fecundity, metabolism, and oxidative stress. For some phenotypes, there was little to no difference between the two sugars. Lifespan, feeding rate, and fecundity varied very little between the two sugars when compared at equal concentrations of up to 20% weight/volume [35] although high sugar concentration phenotypes differed from low sugar and were more severe. Both glucose and fructose similarly increased protein thiols and reduced lipid peroxides as sugar content increased from 0.25% to 10% [36]. Yet high glucose preferentially reduced mitochondrial respiratory capacity, whereas high fructose increased peroxide production, suggesting that these sugars differentially regulate catabolism [36]. Some mitochondrial phenotypes were even sex-specific [36]. In another study, the authors found that a 20% high-glucose diet led to more severe developmental delay and lethality along with hyperglycemia, compared with 20% high-fructose [34]. In young adult flies, obesity, glycogen, and trehalose accumulation were all higher, by contrast, on 20% fructose fed [34]. In our study, we found few differences comparing the CFD with CGD or the HFD with HGD. It is possible that the concentrations used overwhelmed metabolic or stress pathways and that intermediate concentrations would have revealed more monosaccharide-specific significant differences. It may also be that most fructose is converted to glucose in larvae: RNA-seq studies showed high phosphoglucose isomerase expression in the larval gut, which easily converts fructose to glucose (via their phosphorylated forms) and vice versa, depending upon substrate concentrations. It may also be that metabolic effects of fructose are more deleterious than glucose during larval development, but the effects are delayed or not apparent until young adulthood. Although high-sugar diets have reduced longevity in several studies, in a study where glucose feeding was increased more modestly (adding 10% glucose to a 1.7% sucrose diet), it led to increased adult longevity [38]. Considering the complex and numerous fates of dietary sugar, it is not surprising that the effects of fructose and glucose overload seem to depend upon genotype, sex, developmental stage, concentrations, and dietary contexts. We regularly observe significant differences among various control genetic backgrounds, and our control flies were of a different genotype (*w*^*1118*^ outcrosses) than those studied previously [the common *Canton-S* [34,36], a wild-type *IF* line [35,37], and another wild-type line derived from a single wild-caught female from the Vancouver area [38]]. Taken together, these studies are consistent with models where the effects of high-calorie diets depend upon genetic susceptibility and also on the balance between sugars and other dietary components.

Although thousands of DE genes were detected between control and high-sugar diets, very few DE genes could be associated with each sugar. Surprisingly, there was no overlap between the control sugar-dependent and high sugar-dependent DE genes (Fig 5B). Remarkably, only one gene (*CG6602*) differed between the HFD and HGD, and this gene seemed to play little role in the phenotypes tested. Several genes that were differentially expressed in CFD vs CGD were interesting, with an overlap between monosaccharide-dependent genes and insulin receptor-dependent genes [24]. The first of these, *Sgs7*, is a poorly-understood nuclear hormone receptor target gene, and another, *IM3*, encodes an immune response peptide [39]. A third monosaccharide- and insulin-dependent gene, *CG42798*, has no described alleles or functions. Not much is known about the roles these genes might play in metabolism and we could find no mammalian orthologs for *Sgs7*, *IM3*, *CG42798*, or *CG6602*, making them weak candidates for future study. Taken together, our data suggests that *Drosophila* may be of use as a model in which to study the effects of fructose and glucose on metabolism and insulin signaling.

## Materials and methods

### *Drosophila* stocks used

*UAS-Dcr2* [31] was combined with *r4-GAL4* [32] to express UAS-dependent transgenes in the fat body. Other stocks were from the Vienna Drosophila Resource Center (VDRC): lines 60000 (*w*^*1118*^, the GD line control), 60100 (KK insertion site control), and the UAS-dependent RNAi lines *CG6602i*^*18900*^ (GD 18900) and *CG6602i*^*106152*^ (KK 106152).

### Fly husbandry and diet preparation

Stocks were maintained on a standard cornmeal-yeast-agar medium. For experiments, a modified Bloomington semi-defined medium was used [5] containing either 5.4% w/v fructose or glucose (0.3 M, control diets) or 30.6% w/v fructose or glucose (1.7 M, high-sugar diets) for all of the sugar. Flies were reared at 25°C on a 12 hour light, 12 hour dark cycle.

### Wet weights

Wandering third instar larvae were collected, rinsed thoroughly with PBS, dried on a Kimwipe, and weighed in groups of six in a 1.5 ml tube using an analytical balance.

### Triacylglyceride assays

Wandering third instar larvae were frozen in groups of six and then homogenized and assayed as described previously [5]. Briefly, animals are homogenized in PBS + 0.1% Tween, then heated at 65°C for 5 minutes to inactivate lipases. After cooling to room temp, homogenates are vortexed then added to Infinity Triglyceride assay reagent (ThermoFisher TR22421) and incubated at 37°C for five minutes. Absorbance is quantified at 540 nm against a standard curve.

### Hemolymph glucose assays

Wandering third instar larvae were collected and rinsed, then hemolymph was collected and assayed as described previously [5]. Briefly, fine forceps are used to wound the animal and 6–10 larvae are combined to isolate 1–2 microliters of hemolymph. This is added to frozen Infinity Glucose Hexokinase assay reagent (ThermoFisher TR15321), then defrosted once all samples have been added and incubated for 5 minutes at 37°C. Absorbance is quantified at 340 nm against a standard curve.

### Western blotting

Wandering third instar fat bodies were treated with 1 μM insulin in Schneider’s Insect Medium (both from Sigma) for 15 minutes, then fat bodies were harvested and boiled in 2x sample buffer. Approximately 3–5 fat bodies were loaded per lane. Blots were blocked and probed in TBS-T + 5% milk and incubated with primary antibodies overnight at 4°C. Antibodies #8C3 (DSHB, 1:5000) and #4054 (Cell Signaling, 1:500) were used to detect syntaxin (loading control) and PO_4_-Akt (at Ser505), respectively.

### RNA-seq

Three biological replicates were used for each diet. Wandering third instar females were collected between 10:00 and 14:00 each day, inverted, and fat bodies isolated in 1X PBS by a combination of fine dissection and centrifugation at 15,000 x for 1 minute. Females were used because they can be prepared more quickly, have larger fat bodies, avoid the potential mistake of testes in the sample, and because some genotypes are male lethal on high-sugar diets. After fat body purity was checked by microscope, fat bodies were frozen in Tripure (Roche), then extracted and purified using chloroform, isopropanol, and Qiagen’s RNeasy columns. Ribo-Zero and library preps, sequence mapping, and differential expression analyses were done by Washington University’s Genome Technology Access Center. The samples were aligned with *D*. *melanogaster* reference genome, build Ensembl_R72, using TopHat version 2.0.9 and Bowtie 2.0, and the genes were quantitated with HTSeq version 0.5.4 (S1 Table). To select statistically significant differentially expressed genes, we used EdgeR using the negative binomial model exact test with tag wise dispersions (without a Bayesian approach) and a false discovery rate (FDR) cutoff of 5% [40]. Full raw data can be found at the NIH’s Gene Expression Omnibus (GEO) dataset GSE121059. (https://www.ncbi.nlm.nih.gov/geo/query/acc.cgi?acc=GSE121059) DAVID was used to compare differentially expressed gene sets and enriched gene ontology categories were selected as those over-represented with a p < 0.05 [41].

## Supporting information

S1 Table RNA from wandering third instar larval fat bodies from larvae reared on 0.3 M fructose (0.3MF), 0.3 M glucose (0.3MG), 1.7M fructose (1.7MF), or 1.7M glucose (1.7MG) was used for Illumina Hi-Seq based RNA-seq and aligned to the Drosophila melanogaster reference genome version Ensembl_R72 to produce the sequence data shown below. Three biological replicates were used for each sample type.
(DOCX)
