# Supplementary material for: Similar effects of high-fructose and high-glucose feeding in a Drosophila model of obesity and diabetes
Source: PLoS One. 2019 May 15;14(5):e0217096. doi: 10.1371/journal.pone.0217096 (PMC6519815; doi:10.1371/journal.pone.0217096)
Supplement: S1 Table — Three biological replicates were used for each sample type. (DOCX) [file pone.0217096.s001.docx]

**S1 Table.** RNA from wandering third instar larval fat bodies from larvae reared on 0.3 M fructose (0.3MF), 0.3 M glucose (0.3MG), 1.7M fructose (1.7MF), or 1.7M glucose (1.7MG) was used for Illumina Hi-Seq based RNA-seq and aligned to the *Drosophila melanogaster* reference genome version Ensembl_R72 to produce the sequence data shown below. Three biological replicates were used for each sample type.

|  | **0.3MF-1** | **0.3MF-2** | **0.3MF-3** | **0.3MG-1** | **0.3MG-2** | **0.3MG-3** | **1.7F-4** | **1.7F-5** | **1.7F-6** | **1.7MG-1** | 1.7MG-2 | 1.7MG-3 |
| --- | --- | --- | --- | --- | --- | --- | --- | --- | --- | --- | --- | --- |
| *Reads* | 15,416,808 | 16,085,448 | 16,133,717 | 15,249,267 | 14,919,137 | 15,450,399 | 16,037,165 | 15,032,751 | 16,413,242 | 17,136,017 | 16,720,541 | 14,759,695 |
| *Total mapped reads* | 14,398,572 (93.40%) | 15,152,861 (94.20%) | 15,281,932 (94.72%) | 13,907,012 (91.20%) | 13,933,250 (93.39%) | 14,675,083 (94.98%) | 15,059,576 (93.90%) | 14,571,543 (96.93%) | 13,233,415 (80.63%) | 14,808,070 (86.41%) | 14,170,244 (84.75%) | 13,670,467 (92.62%) |
| *Genes detected* | 11,850 | 11,868 | 11,964 | 11,998 | 12,060 | 11,655 | 11,542 | 10,296 | 11,221 | 11,012 | 11,380 | 11,402 |
